# Soil Texture Mediates the Toxicity of ZnO and Fe_3_O_4_ Nanoparticles to Microbial Activity

**DOI:** 10.3390/toxics13020084

**Published:** 2025-01-24

**Authors:** Ghulam Mustafa Shah, Zunaira Shabbir, Faiz Rabbani, Muhammad Imtiaz Rashid, Hafiz Faiq Bakhat, Muhammad Asif Naeem, Ghulam Abbas, Ghulam Abbas Shah, Naeem Shahid

**Affiliations:** 1 Department of Environmental Sciences, COMSATS University Islamabad, Vehari-Campus, Vehari 61100, Pakistan; cr7.zuni@gmail.com (Z.S.); faizrabbani@cuivehari.edu.pk (F.R.); faiqsiddique@cuivehari.edu.pk (H.F.B.); asif.naeem@cuivehari.edu.pk (M.A.N.); 2 Center of Excellence in Environmental Studies, King Abdulaziz University, P.O. Box 80216, Jeddah 21589, Saudi Arabia; irmaliks@gmail.com; 3 Department of Biosciences, COMSATS University Islamabad, Islamabad 45550, Pakistan; ghulam.abbas@comsats.edu.pk; 4 Department of Agronomy, University of Agriculture, Faisalabad 38000, Pakistan; shahjee1522@gmail.com; 5 System-Ecotoxicology, Helmholtz Centre for Environmental Research—UFZ, Permoserstraße 15, 04318 Leipzig, Germany

**Keywords:** soil texture, nanoparticles, ecotoxicity, microbial parameters, soil functions, waste reutilization

## Abstract

The widespread use of metal oxide nanoparticles (NPs) in industrial and household products has raised concerns about their potential soil contamination and its ecological consequences. The purpose of this study was to examine and compare the effects of iron oxide nanoparticles (FeONPs) and zinc oxide nanoparticles (ZnONPs) on the microbial activity and biochemical properties of differently textured soils. A mesocosm experiment was conducted using three soil types–clay loam (CL), sandy clay loam (SCL), and sandy loam (SL) amended with farmyard manure (FYM), ZnONPs and/or FeONPs. The results revealed significant differences in microbial colony-forming units (CFUs) and carbon dioxide (CO_2_) emissions in the order of SL > SCL > CL. Compared with those from the unfertilized control, the CO_2_ emissions from the FYM increased by 112%, 184% and 221% for CL, SCL and SL, respectively. The addition of ZnONPs and FeONPs notably increased the microbial biomass Zn/Fe, which reflected their consumption by the soil microbes. As a result, microbial CFUs were considerably reduced, which led to a 24%, 8% and 12% reduction in cumulative CO_2_ emissions after the addition of ZnONPs to the CL, SCL and SL soils, respectively. The respective decrements in the case of FeONPs were 19%, 2% and 12%. The temporal dynamics of CO_2_ emissions revealed that the CO_2_ emissions from CL with or without FYM/NPs did not differ much during the first few days and later became pronounced with time. Almost all the studied chemical characteristics of the soils were not strongly affected by the ZnONPs/FeONPs, except EC, which decreased with the addition of these nanomaterials to the manure-amended soils. Principal component analysis revealed that the ZnONPs and FeONPs are negatively corelated with microbial CFUs, and CO_2_ emission, with ZnONPs being more toxic to soil microbes than FeONPs, though their toxicity is strongly influenced by soil texture. Hence, these findings suggest that while both these NPs have the potential to impair microbial activity, their effects are mediated by soil texture.

## 1. Introduction

Nanoparticles (NPs) are the smallest particles with dimensions of 1–100 nm and are composed of materials such as metals, metal oxides, polymers, etc. [1]. The NPs are chemically responsive, mechanically strong and have a large surface area. These unique properties enable them to be a suitable material to use in various industrial applications [1]. Therefore, NPs use has tremendously increased in various sectors, such as nuclear, environmental remediation, astronomy, pharmaceutical [2], energy, wastewater treatment, agriculture and healthcare [3]. The global production and use of NPs between 2010 and 2014 ranged from 225,060 metric tons to approximately 585,000 metric tons [4,5]. NPs may be deposited into soil through various pathways, at each step from their production to applications. Modern agricultural practices such as soil remediation or coated fertilizers directly introduce the NPs into the terrestrial environment [6,7]. Further, NPs can reach soil via the application of activated sludge, industrial or domestic waste containing trace elements of metal oxide NPs that are applied as soil conditioner/fertilizer [8]. In soil, NPs interact with soil microbes and can be potentially toxic for microbes and thereby the soil health [9]. These effects are clear in previous reports that suggested the growth of soil microbes is adversely affected by the NPs, as they serve as growth inhibitors [10,11], and resulted in toxic effects on colony-forming units, enzymatic activities and microbial mass [5]. These interactions can greatly interfere with crucial microbial processes like nutrient cycling and organic matter decomposition [12,13]. The toxicity mechanism of NPs is mainly associated with the induction of oxidative stress, damage to biological membranes and microbial metabolic pathways [14].

Historically, the metal oxide NPs have been used as biocides to curtail the microbial growth [5]. Therefore, after soil application, Rashid et al. [11], observed reduction in microbes-mediated processes, i.e., decomposition and N mineralization from leaf litter by approximately 130% and 122%, respectively, in the presence of zinc oxide NPs. Likewise, these processes were reduced by 60% from grass litter in the presence of iron oxide NPs [11]. Kamran et al. [15] also observed a decrease of up to 28 and 35% in microbial decomposition and organic N mineralization, respectively, in manures amended soil mixed with iron oxide NPs. In another study, Shah et al. [16] found a reduction in microbial CFUs by 32 and 19% after the application of poultry manure to soil contaminated with ZnONPs and FeONPs, respectively. In the respective scenarios, 62 and 29% lower microbial activities, i.e., mineralization had occurred. The toxic effect can be different according to the type of nanoparticle, the concentration of the nanoparticle and the soil properties, such as soil texture and organic matter content [17,18,19]. These coexistences may control the ecological risks of NPs in agronomical and environmental management; hence, understanding them is crucial.

Metal oxide NPs interact with microbial and faunal communities in a soil-specific manner [20]. When entering the soil, the NPs interact with the components of the solid phase such as soil organic matter (SOM), clay particles and aggregates by various processes which include adsorption, cation exchange and fixation processes [21]. Such interactions decrease the mobility of the NPs in the soil matrix and thus curtail their influence on soil organisms and their functions [22]. Further, physicochemical characteristics of NPs including size, chemical composition, surface charge, and environmental factors like soil texture, pH and ionic strength reduce the extent of NPs sorption and mobility [23]. NPs, being smaller in size, are highly mobile and can move faster than larger particles before being trapped in the soil matrix. The sorption intensity of NPs can vary because of their size, chemistry, accumulation and environmental conditions for their application [24]. The presence of a solid phase further complicates the fate of NPs in soil systems. In the solid phase, the fate of NPs can be affected by the charged components of soil, e.g., clay particles or the presence of humic substances. In an aqueous phase, NPs would interact with colloids like humic substances and a large variety of ions/cations and thus affect the NPs stability, solubility and bioavailability. Such interactions may greatly mediate NPs behaviors, which may influence their aggregation, dissolution, and mobility [14]. Aromatic compounds and functional groups in the soil solution, for instance, have been shown to adsorb onto NPs surfaces and thus may further influence their stability in a soil environment [24].

Either from the solid or aqueous phase, these metal oxide NPs when exposed to soil microorganisms result in direct toxicity, i.e., microbial membrane disruption, ROS formation or genotoxicity, leading to cell death, and thereby the cultivable microbial colony forming units decline. Such toxic effects have been reported in earlier studies; i.e., Palza [25] reported the decline of various fungal and bacterial species due to the toxic effects of metal oxide NPs. Chai et al. [26] found a decrease in azotobacter together with their enzymatic activities in response to the toxicological effects of zinc oxide NPs. Chen et al. [27] found inhibition in microbial decomposition, mineralization and enzymatic (hydrolase, and dehydrogenase) activities after soil application of ZnONPs. As a result of these toxicological activities from NPs, microbial-mediated carbon and nitrogen cycling from soil-applied organic material/manures can be severely hampered.

The addition of organic materials such as farmyard manure, compost or leaf litter to soil is essential for better carbon sequestration and the availability of plant nutrients in agro-ecosystems. However, once these organic materials are applied to soil, microbe-mediated decomposition and mineralization are needed which is particularly complex and are usually controlled by a variety of factors, such as soil texture, microbial activity and the presence of pollutants, i.e., nanoparticles [12,28,29,30]. Soils with higher clay content tend to show reduced decomposition and mineralization rates due to the fixation of organic material in interlayer spaces of clay minerals [31], the entrapment of added material within the soil aggregates thus restricting the access of soil microbes, and the physical protection of microbial biomass within the soil structure [30]. For instance, Shah et al. [29] reported that soils with greater clay content decrease organic matter decomposition, leading to slower mineralization. Given the potential toxicity of metal oxide NPs to soil microbes [13,16], it is critical to understand how these particles influence the microbial-mediated decomposition and mineralization processes across different soil types. Although, research on the environmental impact of NPs on microbial activities and soil functions is limited, most studies have focused on plant litter decomposition [11] or animal manure [13,15,16] applied to a single type of soil. To date, there is a lack of comprehensive data on the fate of metal oxide NPs like ZnONPs and FeONPs in different soil types. Understanding these interactions is crucial for assessing the broader ecological risks posed by NPs in agricultural soils. The aim of this study was to (i) estimate and compare the effects of FeONPs and ZnONPs on microbial activity and biochemical properties of different texture soils, and (ii) the role of soil texture on the toxic effects of FeONPs and ZnONPs.

## 2. Materials and Methods

A mesocosm experiment was carried out indoors using plastic jars at COMSATS University Islamabad, Vehari Campus (30.0318° N, 72.3145° E), Pakistan. Soils were collected from identified fields in the district of Vehari. Farmyard manure (FYM) was procured from a nearby agricultural farm. The characteristics of the soil and farmyard manure are provided in Table 1 and Table 2, respectively. Iron oxide nanoparticles (FeONPs, Fe_3_O_4_) and zinc oxide nanoparticles (ZnONPs, ZnO) were in powder form and obtained from Sigma-Aldrich (Saint Louis, MO, USA). As per the supplier information, FeONPs exhibited a particle size of ≤50 nm and a particle density of 5.25 ± 0.1 g/mL. For ZnONPs, the corresponding values were ≤40 nm and 5.67 ± 0.1 g/mL. The ZnONPs used in these experiments were from the same batch as used in Shah et al. [16].

### 2.1. Characterization of the Metal Oxide Nanoparticles

The abovementioned ZnONPs and FeONPs were examined for surface morphology by scanning electron microscopy (SEM, S-4700, Hitachi, Chiyoda City, Tokyo, Japan). The surface of each sample was gold-sputtered (250 Å) via a Jeol JFC-1500 anion sputtering machine (Japan 196–8558). The surface morphologies of the ZnONPs and FeONPs were determined via a secondary electron detector at a voltage of 20 kV and a magnification range of 25x–50kx. An energy-dispersive X-ray (EDAX) instrument coupled with SEM was used to determine the elemental composition of the ZnONPs and FeONPs samples. The FTIR spectrum of the NPs was obtained via a Fourier transform infrared (FTIR) spectrometer (PerkinElmer, 710 Bridgeport Avenue, Shelton, CT 06484–4794, USA). To prepare this mixture, a dry powder of ZnONPs and FeONPs was mixed with 10 mg of dry KBr powder and dried to remove any moisture. For IR, the powder was analyzed at 500–4000 cm^−1^.

### 2.2. Experimental Setup and Treatments

**Figure 1:**
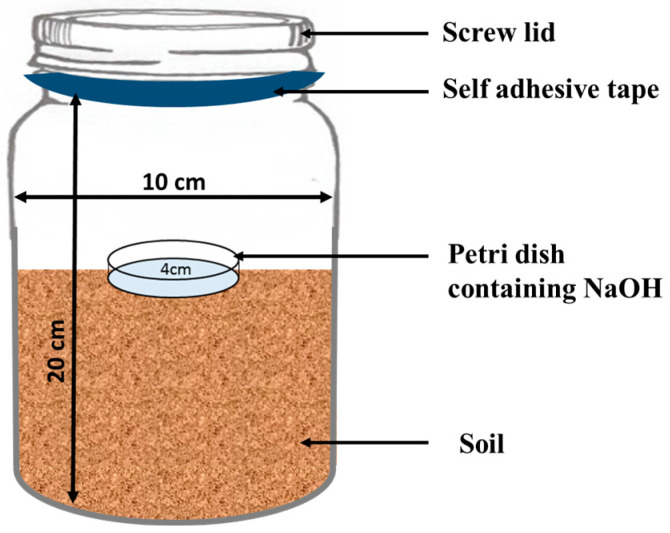
Schematic diagram of the experimental setup for the implementation of the experiment.

In the laboratory experiment, 1 L plastic jars were filled with 500 g of moist soil (Figure 1). The treatments were subsequently applied to the three soil types (clay loam, sandy clay loam and loam soil) as per plan. For each of the three soil types, the treatments included were (i) unfertilized soil as a control, (ii) ZnONPs, (iii) FeONPs, (iv) FYM, (v) FYM + ZnONPs and (vi) FYM + FeONPs. All the treatments were replicated thrice and were arranged in a completely randomized design. ZnONPs and FeONPs were applied to the soil at a proportion of 0.5 g per kg of soil. Approximately 10 g of dried and crushed FYM was applied to the jars. Using a spatula, the organic waste and the NPs were mixed in the soil soon after their application. During the experimental period of 90 days, the moisture content of the jars was maintained at 60%. The moisture content in the jars was periodically maintained via a digital moisture meter, and moisture loss was replenished with distilled water when necessary. The jars were placed at room temperature (25 ± 1 °C) for 90 days.

### 2.3. Measurement of CO_2_ Emissions

In a small Petri dish, 10 mL of NaOH (1 M) solution was added, and the mixture was placed in a 1 L plastic jar containing various treatments to capture the emitted CO_2_ from the soil. To avoid CO_2_ escape, the plastic jars were sealed with a screw lid which was then firmly wrapped with a self-adhesive tape (Figure 1). The release of CO_2_ was measured on days 2, 5, 7, 15, 30, 45, 60 and 90 of the experiment. At each sampling event, the Petri dishes were taken out from the jars and replaced with new ones containing freshly prepared NaOH solution. Each of the sampled NaOH solutions was back-titrated against HCL (1 M) as reported in Kamran et al. [15]. CO_2_ emissions were calculated via the following equation. The CO_2_ emission after each sampling event was summed to calculate cumulative CO_2_ emission.

$${CO}_{2}=0.5\times \frac{V_{NaOH}{\;\times \;C}_{NaOH}}{1000}-\frac{V_{HCL}\times C_{HCL}}{1000}\times 100\times 44\times 1000$$

where ${CO}_{2}$ is the carbon dioxide emission from soil, ${C_{NaOH}andV}_{NaOH}$ represents the concentration and volume of NaOH place in Petri dishes, respectively, $V_{HCL}$ and $C_{HCL}$ represents the volume and concentration of HCL used during back-titration.

### 2.4. Soil Sampling and Analysis

At the end of the experiment, representative soil samples were taken from each jar and analyzed for various selected biochemical properties. The pH was determined in an extract of soil and KCL at a 1:10 ratio using a calibrated pH meter. The same extract was used to determine EC contents with an EC meter. The organic matter content of the soil was determined using a muffle furnace, combusting samples at 525 °C for 6 h, through the loss on ignition method as reported by [32], and the total organic C was calculated following the procedure reported in Estefan [33]. Soil mineral N (NH_4_-N + NO_3_-N) contents as well as total N contents were determined using the Kjeldahal method. The fumigation–extraction method reported in Estefan [33] was used to determine microbial biomass Fe/Zn contents. For this purpose, the soil samples were fumigated with chloroform and extracted with a potassium sulfate solution. The Zn and Fe contents of the extracts were analyzed via an atomic absorption spectrophotometer (AAS, Model Thermo S-Series, Thermo Fisher Scientific, Waltham, MA, USA). To determine bacterial and fungal colony counts, nutrient agar plates were inoculated with soil suspensions and incubated at 37 °C for 24–48 h. Colonies were counted via a digital colony counter (ColonyCount V, Gerber Instruments AG, Effretikon, Switzerland) to calculate the number of colonies forming units (CFUs) per milliliter.

### 2.5. Statistical Analysis

The collected data were statistically analyzed via analysis of variance in STATISTIX 8.1 The treatment means were further compared using the least significant difference (LSD) test at the 5% probability level. The impacts of ZnONPs, FeONPs and FYM on soil microbial biomass Fe/Zn, microbial colony forming units, CO_2_ emission, EC, pH, OM and TOC, as well as their correlations, were evaluated via principal component analysis (PCA) in CANOCO 5.0 for Windows (Microcomputer Power 281 Inc., Ithaca, NY, USA) on correlation matrices.

## 3. Results

### 3.1. Characterization of the Nanoparticles

**Figure 2:**
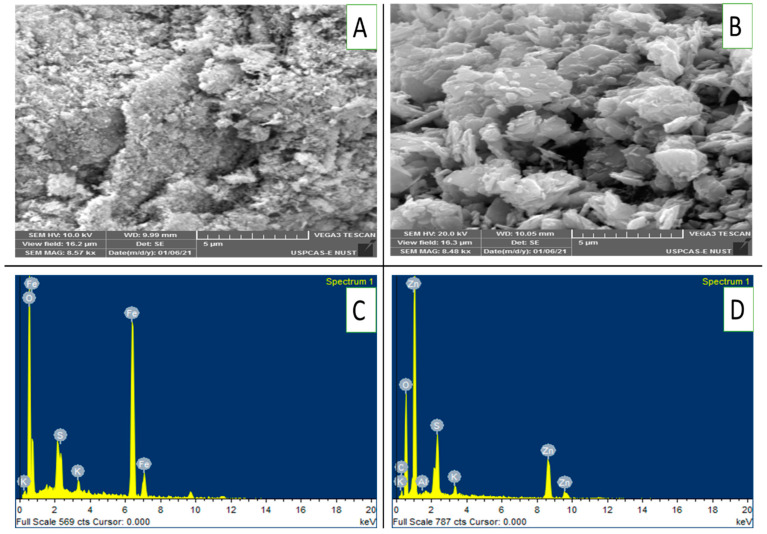
(**A**–**D**): SEM images and EDX patterns of FeONPs and ZnONPs: (**A**) SEM image of FeONPs, (**B**) SEM image of ZnONPs, (**C**) EDX pattern of FeONPs and (**D**) EDX pattern of ZnONPs. ZnONPs used in this study were from the same batch as that from one of our previous work [16].

Figure 2A,B presents scanning electron microscopy (SEM) images of FeONPs and ZnONPs, revealing their surface morphology at a magnification of 8.5kx. Figure 2A clearly shows that the FeONPs are composed of a rough, textured surface with irregular particles. On the other hand, the ZnONPs had a more agglomerated structure with larger, flake-like particles. EDX analysis revealed the presence of Fe, K, S and O on the FeONPs, as depicted in Figure 2C. Similarly, ZnONPs presented peaks for Zn, K, O, S and Al in their EDX spectra (Figure 2D).

**Figure 3:**
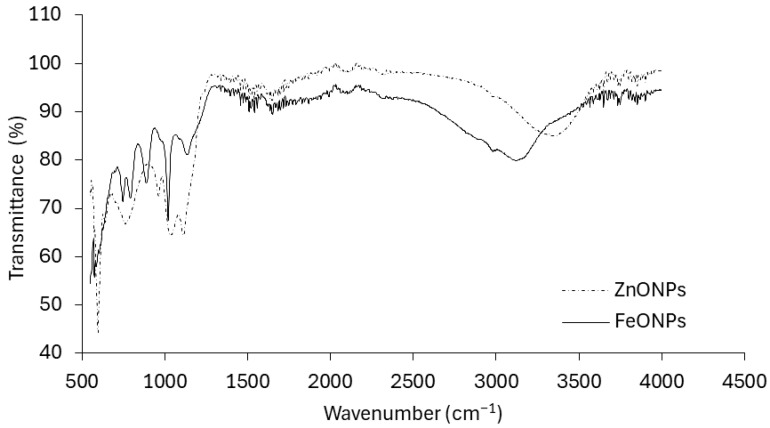
FTIR patterns of FeONPs and ZnONPs used in this study.

These findings provide clear evidence of successful Fe and Zn impregnation onto the respective nanoparticles. Figure 3 presents the infrared (IR) spectra of the FeONPs and ZnONPs. The spectrum of the FeONPs exhibited a complex pattern with multiple peaks and valleys across the wavenumber range. It exhibited a broad absorption band at approximately 3300 cm^−1^, which is characteristic of O-H stretching vibrations. Other prominent peaks are observed at approximately 2900 cm^−1^ (C–H stretching), 1600 cm^−1^ (C=C stretching), and 1000 cm^−1^ (C–O stretching). The spectrum of the ZnONPs appeared simpler than that of the FeONPs, with fewer distinct peaks. It shows a strong absorption band at approximately 1700 cm^−1^, which is typically associated with C=O stretching vibrations. Additionally, there are peaks at approximately 2900 cm^−1^ (C–H stretching) and a broader band at approximately 3300 cm^−1^ (O–H stretching).

### 3.2. Carbon Dioxide Emission

**Figure 4:**
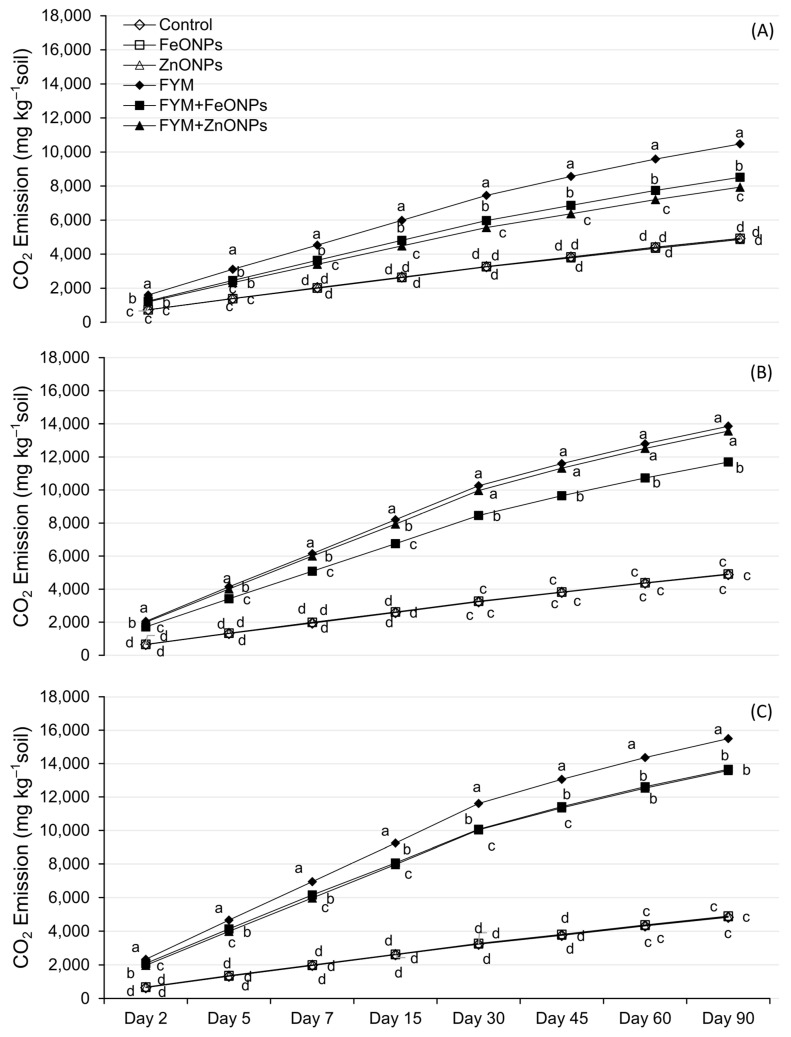
CO_2_ emissions from FYM applied to NPs contaminated (**A**) clay loam soil, (**B**) sandy clay loam soil and (**C**) sandy loam soil. Data points, on a given day, carrying different letters are significantly different from each other.

Figure 4A–C shows the temporal dynamics of CO_2_ emission from FYM with or without the addition of ZnONPs/FeONPs in clay loam (CL), sandy clay loam (SCL) and sandy loam (SL). In all the soil types, CO_2_ emissions were unaffected by the addition of ZnONPs or FeONPs to the unfertilized soil (*p* > 0.005). Compared with the control, soil-addition of FYM significantly increased CO_2_ emissions, irrespective of the soil type (*p* < 0.05). However, the addition of ZnONPs or FeONPs to the FYM-amended soil notably reduced CO_2_ emissions compared with the individual application of FYM. This difference was small during the early days of incubation and subsequently increased with increasing incubation time. Especially in the CL soil, the difference among the treatments was almost negligible in the first few days, which later became pronounced after the first week of the incubation period (Figure 4A). For the remaining two soil types, the CO_2_ emissions significantly differed from those on day 2 of the incubation period. Over an incubation period of 90 days, the cumulative emission from FYM with or without the addition of ZnONPs or FeONPs from CL was lower than that from the other materials. For each soil type, there was no significant difference in emissions between unfertilized soil with or without ZnONPs/FeONPs (*p* > 0.05).

**Figure 5:**
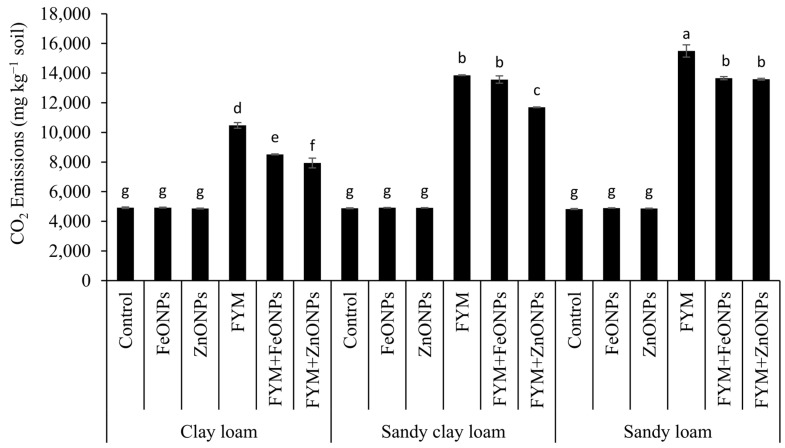
Cumulative CO_2_ emissions from the FYM applied to various soils contaminated with FeONPs/ZnONPs. Bars with different lowercase letters are significantly different from each other at 5% probability.

Compared with those in unfertilized soil, cumulative CO_2_ emissions over the experimental period from FYM were 53% (10,471 vs. 4917 mg kg soil^−1^), 65% (13,857 vs. 4880 mg kg soil^−1^) and 69% (15,493 vs. 4830 mg kg soil^−1^) greater in CL, SCL and SL soils, respectively (*p* < 0.05; Figure 5). The addition of ZnONPs to the FYM-amended soil significantly reduced the cumulative emission by 24% (10,471 vs. 7934 mg kg soil^−1^), 8% (13,857 vs. 12,823) and 12% (15,493 vs. 13,588) in the CL, SCL and SL soils, respectively, but the emission was significantly greater than that in the unfertilized control (only soil). The respective decreases in the case of FeONPs were 19% (10,471 vs. 8516 mg kg soil^−1^), 2% (13,857 vs. 13,566 mg kg soil^−1^) and 12% (15,493 vs. 13,659 mg kg soil^−1^). The soil type had a significant effect on CO_2_ emission, with the highest values in the SL soils and the lowest values in the CL soils, whereas SCL had an intermediate effect (Figure 5).

### 3.3. Soil Chemical Parameters

Soil chemical parameters (pH, OM, TOC, and EC) were assessed after a 90-day incubation period for soils amended with FYM, ZnONPs, and/or FeONPs (Table 3). There was no significant difference in most of the studied parameters due to the addition of metal oxide nanoparticles. While pH, organic matter (OM) and total organic carbon (TOC) content remained relatively consistent across all the treatments and soil types, electrical conductivity (EC) exhibited significant variations. Compared with the unfertilized control, the sole application of FYM, ZnONPs and FeONPs increased the EC, irrespective of the soil type. However, the addition of ZnONPs or FeONPs to the FYM-amended soil led to a notable decrease in EC compared with that in the individual FYM treatments across all the soil types (*p* < 0.05; Table 3).

### 3.4. Microbial Biomass Zn/Fe Contents

**Figure 6:**
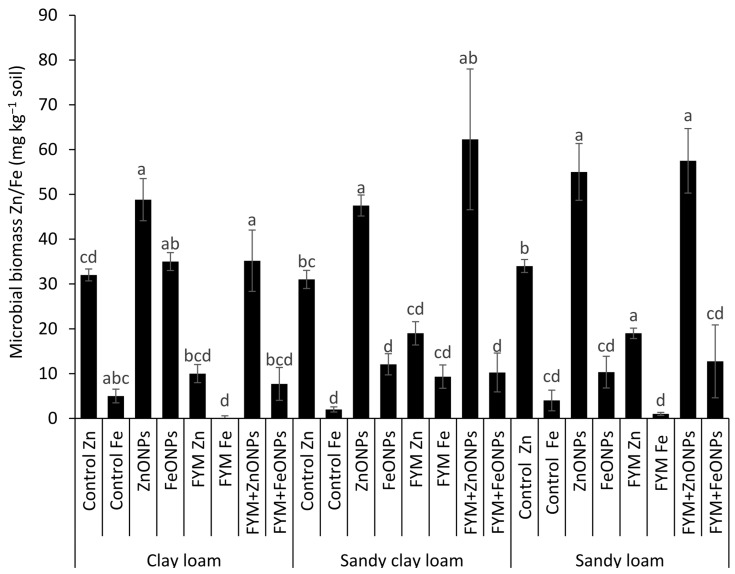
Average (*n* = 3, ±standard errors) microbial biomass Zn/Fe (mg kg^−1^) contents in different soil types. Bars with different lowercase letters are significantly different from each other at 5% probability.

Figure 6 shows the microbial biomass Zn/Fe content from three types of soil (CL, SCL and SL) under different treatments, such as the control, ZnONP, FeONPs, FYM, FYM+ZnONP and FYM+FeONPs. There was a significant difference in the microbial biomass Fe/Ze content between unfertilized soil with or without ZnONPs and FeONPs, irrespective of the soil type, reflecting their consumption by the soil microbes. For example, the addition of ZnONPs to soil significantly increased the microbial biomass of Zn by 54% (32 vs. 49 mg kg^−1^) in CL, 54% (31 vs. 48 mg kg^−1^) in SCL and 61% (34 vs. 0.055 mg kg^−1^) in SL compared with their respective unfertilized controls (*p* < 0.05; Figure 6). The microbial biomass Fe content increased by 600% (5 vs. 35 mg kg^−1^), 500% (2 vs. 12 mg kg^−1^) and 150% (4 vs. 10 mg kg^−1^) in the CL, SCL and SL, respectively (*p* < 0.05). Compared with the control, the sole application of FYM significantly decreased the microbial biomass Zn content by 68% (32 vs. 10 mg kg^−1^) in CL, 38% (31 vs. 19 mg kg^−1^) in SCL and 44% (34 vs. 19 mg kg^−1^) in SL. However, the microbial biomass Fe content after the sole application of farmyard manure was not significantly different from that of the respective unfertilized control for all the soil types. In each soil type, the combined application of FYM with ZnONPs or FeONPs had a significant effect on the microbial biomass Zn or Fe content. For example, the microbial biomass of Zn was increased by 350% (10 vs. 35 mg kg^−1^) and that of Fe was increased by 800% (1 vs. 8 mg kg^−1^) compared with those resulting from the sole application of FYM in CL soil. The respective increases were 200% (19 vs. 57 mg kg^−1^) and 1200% (1 vs. 13 mg kg^−1−1^) in the case of the SL soil. In the SCL soil, ZnONP addition to the FYM-amended soil increased the microbial biomass Zn content by 226% (19 vs. 62 mg kg^−1^), whereas the Fe content was similar (12 vs. 10 mg kg^−1^) to that in the FYM-amended soil alone.

### 3.5. Viable Microbial Colony-Forming Units

**Figure 7:**
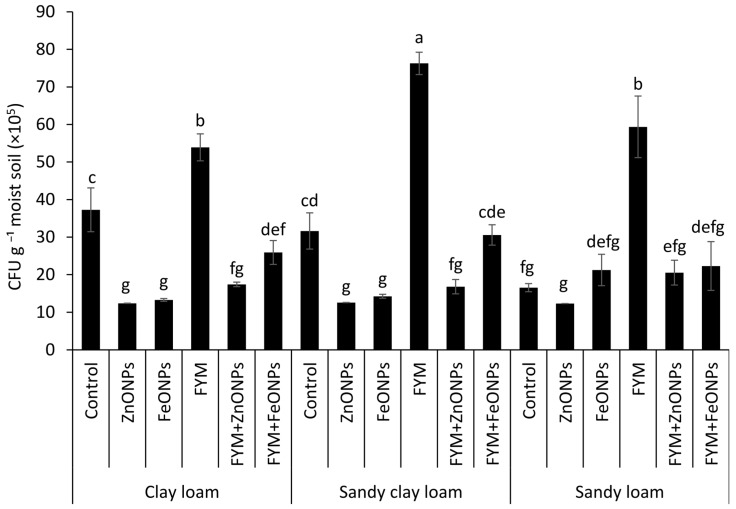
Average (*n* = 3, ± standard errors) number of cultivable colony-forming units (CFU) of bacteria. Bars with different lowercase letters are significantly different from each other at 5% probability.

Cultivable microbial colony forming units (CFUs) from each type of soil (clay loam, sandy clay loam and sandy loam) under different treatments, such as the control, ZnONPs, FeONPs, FYM, FYM+ZnONPs and FYM+FeONPs treatments, are presented in Figure 7. Although the CFU counts of the unfertilized control without ZnONPs or FeONPs were relatively greater than those with ZnONPs or FeONPs, the values were not significantly different among the soil types. The addition of FYM increased the CFU across all the soil types; however, the values were not significantly different than those of the control in the CL soil, although they were significantly different in the SCL and SL soils. Compared with unfertilized soil, FYM-amended soil increased the bacterial CFU by 45, 137 and 247% in CL, SCL and SL soils, respectively. Compared with the individual FYM treatment, the addition of ZnONPs to the FYM-amended soil reduced the number of microbial CFU by 68% (54 × 10^5^ vs. 17 × 10^5^ mg L^−1^ soil) in the CL treatment, 77% (74 × 10^5^ vs. 17 × 10^5^ mg L^−1^ soil) in the SCL treatment and 64% (59 × 10^5^ vs. 21 × 10^5^ mg L^−1^ soil) in the SL treatment. The respective decreases in the case of FeONPs were 51% (54 × 10^5^ vs. 26 × 10^5^ mg L^−1^ soil), 59% (76 × 10^5^ vs. 31 × 10^5^ mg L^−1^ soil) and 62% (59 × 10^5^ vs. 22 × 10^5^ mg L^−1^ soil). Among the soil types, the highest CFU values from the FYM with and/or without NPs were observed in the sandy clay loam, whereas the lowest values were observed in the clay loam (Figure 7). Between the ZnONPs and FeONPs in the FYM-amended soils, the CFU values were 35% (26 × 10^5^ vs. 17 × 10^5^ mg L^−1^ soil), 45% (31 × 10^5^ vs. 17 × 10^5^ mg L^−1^ soil) and 5% (22 × 10^5^ vs. 21 × 10^5^ mg L^−1^ soil) lower from the CL, SCL and SL soils, respectively, indicating greater toxicity of the former nanoparticles.

### 3.6. Principal Component Analysis

**Figure 8:**
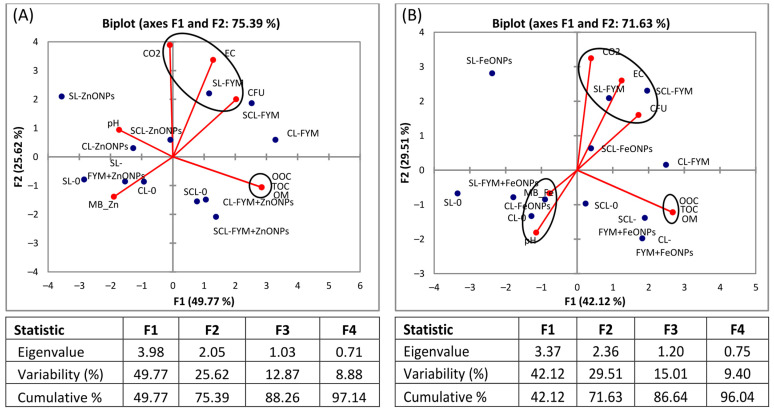
Principal component analysis (PCA) of microbial biomass (MB) Zn/Fe, colony forming units (CFUs) of bacteria, soil pH, EC, OM and TOC after (**A**) application of zinc oxide nanoparticles (ZnONPs) and (**B**) iron oxide nanoparticles (FeONPs) to clay loam (CL), sandy clay loam (SCL) and sandy loam (SL) soils.

The biplot visualizes the relationships between different soil parameters and treatments, as well as the relationships among the soil parameters. The analysis revealed that most of the data variation is accounted for by the first (F1) and second principal components (F2), i.e., 75% and 71% for the ZnONPs and FeONPs, respectively. The PCA results revealed that the sole application of FYM had a positive association with CO_2_ emission, CFU and EC, irrespective of the soil type (Figure 8A,B). However, the microbial biomass Zn/Fe content is strongly negatively correlated with these parameters. In the case of FeONPs, the pH was negatively correlated with CO_2_, CFU and EC (Figure 8B), whereas it was slightly positively associated with CO_2_ in the case of ZnONPs (Figure 8A). On the other hand, OM, TOC and OOC are strongly correlated with each other, irrespective of the soil or nanoparticle type.

## 4. Discussion

We found that the application of FYM to the soil significantly increased CO_2_ emissions compared with those in the untreated control soil (Figure 5). Concurrently, we observed a 2–3-fold increase in the number of bacterial colony-forming units after FYM application (Figure 6), suggesting that the elevated carbon content in the soil resulting from the addition of manure created a conducive environment for microbial proliferation. These results align with those of Yazdanpanah et al. [34], who reported increased soil microbial activity following organic amendment application. Soil bacteria utilize organic matter as a nutrient source for energy, growth, and reproduction. These findings align with previous research demonstrating that incorporating organic matter into soil stimulates microbial population growth [11,35,36]. Consequently, increased microbial activity, as evidenced by increased CO_2_ emissions–a direct indicator of microbial respiration–was observed. Similarly, Rashid et al. [11] reported increased CO_2_ emissions following the application of grass leaf litter to sandy loam soil. Rahman et al. [37] reported increased CO_2_ emissions following the application of livestock dung to agricultural land.

However, CO_2_ emissions were notably lower when ZnONPs or FeONPs were applied to the FYM-amended soil. Our FTIR results suggested that these metal oxide nanoparticles (NPs) had highly reactive surface sites that can bind to soil organic matter (Figure 3). Organic molecules such as humic and fulvic acids present in soil can increase the bioavailability of NPs to soil microbes [38,39]. We detected the Zn and Fe contents within the microbial biomass, indicating that their acquisition by the soil microbes (Figure 6) led to a decline in the number of microbial colony-forming units. These findings corroborate those of Rashid et al. [11], who reported an 84% decrease in the number of bacterial colony-forming units in grass litter-amended soil treated with NPs compared with that in grass litter alone. Similarly, in one of our previous studies, we reported 32 and 43% decreases in bacterial CFUs in FYM- and PM-amended soils, respectively [13]. This led to reduced microbial activity, including the decomposition of FYM. Metal oxide NPs can inhibit microbial activity through several mechanisms: (i) NPs release reactive oxygen species (ROS) that disrupt the cell wall, DNA and cellular proteins resulting in oxidative stress and thereby the microbial functions are impaired, (ii) the release of metal ions (i.e., Zn^2+^ and Fe^2+^) from NPs has the ability to rupture the cell membrane and enter the cellular contents as observed in *E. Coli* by [40], and (iii) being smaller in size, NPs may penetrate the cell wall and internalized into cellular components affecting its micro-climate leading to cell damage. Due to these possible mechanisms, microbial activities, i.e., decomposition and mineralization, are impaired. Consequently, in our study, NPs significantly reduced CO_2_ emissions from the FYM. These results are in line with studies by Kamran et al. [15], Frenk et al. [41] and Rashid et al. [11], which reported decreased CO_2_ emissions from organic matter following NP application. These authors attributed this reduction in CO_2_ emissions to the reduced microbial populations.

In addition to NPs, the soil type influenced the microbial CFUs and their activity, as the CO_2_ emissions from the FYM, with or without NPs, were highest in sandy loam and lowest in clay loam soils (Figure 3). The lower microbial activity in clay soil is likely due to its relatively higher clay content, which (i) tends to have poor drainage and aeration, thus creating anaerobic conditions in soil and thereby reduced microbial activity, as many microorganisms require oxygen for their metabolic processes; (ii) physically entraps the added organic matter in micropores or adsorbs on clay particles, thus limiting their access to the soil microbes, which in turn slows decomposition; and (iii) hinders the movement of microorganisms by their dense structure and lower pore size, limiting their access to the added organic material [29]. Our results are in line with those of Angst et al. (2021), who reported two-fold lower CO_2_ emissions from added organic matter in clayey soil than in sandy soil. Similarly, Shah et al. [29] reported lower decomposition and mineralization of added organic matter in clayey soils than in sandy and peat soils, which they attributed to the greater clay contents. In addition to the clay contents, the soil structure is involved in the decomposition of the added material, as it may affect the distribution pattern of soluble organic compounds within the soil profile. We argue that after the application of FYM to SCL and SL soils, soluble organic compounds were transported much deeper in the soil profile with the mass movement of irrigation water and later through diffusion, with relatively less adsorption of organic matter, thus increasing the opportunity for interaction with soil microbes. This would not have been the case for CL, as the water permeability of soil with greater clay content is usually lower because of its small pore size, dense structure and aggregation; thus, the organic matter compounds and the NPs remained on the top layer. As a result, the toxicity of ZnONPs/FeONPs to soil microbial activities was more pronounced in CL soil than in SCL and SL soils, as CO_2_ emissions were reduced by 2–3-fold after ZnONP application to FYM-amended soil. Similar results were obtained for the FeONPs, where the CO_2_ reduction was 19% in the CL, 2% in the SCL and 12% in the SL soils. These findings indicate that the soil texture can affect the toxicity of both types of metal oxide NPs.

### 4.1. Effects of Metal Oxide Nanoparticles on Soil Chemical Properties

The application of ZnONPs and FeONPs did not affect the soil’s chemical characteristics, such as pH, OM and TOC (Table 3). This may be due to the suitable solubility of the NPs that may not had an effect on the chemical properties of the soil. Our findings also support those of [11], who found in a study that when applying NPs with organic waste similar chemical soil properties were evaluated. However, in the control soils, the EC values increased with the application of ZnONPs and FeONPs, which is less common but can occur under specific conditions. Potential causes include the release of soluble salts from the NPs, disruptions to the soil structure leading to salt accumulation, interactions with soil minerals that liberate ions and the exacerbation of existing salinity issues in manure-amended soils. However, the overall impact on EC is influenced by various factors, including the nanoparticle concentration, soil type, and environmental conditions. Interestingly, the addition of ZnONPs reduced the soil EC of the FYM-amended soil. This could be due to the increase in the soil cation exchange capacity caused by the added NPs, leading to increased adsorption of cations and reduced salt availability. Second, they may also precipitate soluble salts directly, thus lowering their concentration in the soil solution. Additionally, interactions of NPs, organic matter and soil microbes might affect nutrient cycling and salt dynamics leading to a lower EC [42].

### 4.2. Toxicity of Zinc Oxide and Iron Oxide Nanoparticles

Among the NPs, ZnONPs were relatively more toxic to soil microbes than FeONPs, irrespective of the soil type. This was evident in relatively lower bacterial counts together with lower microbial activities, i.e., CO_2_ emission from the former than from the latter NPs. This could be due to differences in their physicochemical characteristics, i.e., size shape, chemical composition and surface chemistry, which determine their toxicity [43]. ZnONPs have high solubilities enabling them to release zinc ions into the soil therefore enhancing bioavailability to microorganisms. Besides that, the surface morphology of the NPs exhibited ZnONPs agglomeration due to high concentration, as evident from the SEM images (Figure 2A), which might lead to a high toxicity to soil microorganisms. This corroborates a previous report by Murugadoss et al. [44] who observed a higher degree of cytotoxicity and DNA damage with nanoparticles of higher agglomerates. Furthermore, the higher surface reactivity of ZnONPs resulted in better interaction with microbial cell components. We suggest that all the above-stated factors might have led to the higher toxicity of ZnONPs as compared to FeONPs in the soil environment.

## 5. Conclusions

Here, we showed for the first time that soil texture can modulate the toxicity of zinc oxide (ZnONPs) and iron oxide nanoparticles (FeONPs) to soil microbes. The present study highlighted the pronounced toxicity exhibited by clay loam (CL) compared to sandy clay loam (SCL) and sandy loam (SL) soils since CO_2_ emissions were reduced by 2–3-fold after ZnONP was applied to FYM-amended CL soil in comparison to its counterparts; SCL and SL soils. Similar results were obtained for FeONPs, where the CO_2_ reduction rates were 19%, 2% and 12%, respectively. This decrease was accompanied by lower bacterial colony-forming units in the CL soil than in the other soils. Among the nanomaterials, ZnONPs were more toxic than FeONPs, irrespective of the soil type. However, no evidence of their impact on soil chemical properties has been reported. Therefore, for better soil health and carbon as well as nitrogen cycling, the use of these metal oxide nanoparticles must be restricted so that they may not end up in the soil, especially in those with greater clay contents.

## Figures and Tables

**Table 1:** Mean (n #3) characteristics of the soils used in this study.

Parameters	Units	Clay Loam	Sandy Clay Loam	Sandy Loam
pH_KCL_ 1:10	-	7.3 (±1.0) *	7.3 (±0.18)	7.1 (±0.90)
EC_KCL_ 1:10 Sand Silt Clay	dS m^−1^ % % %	3.06 (±0.76) 40 (±2.16) 30 (±2.50) 30 (±9.82)	2.86 (±0.29) 52 (±3.52) 28 (±5.72) 20 (±1.33)	2.4 (±0.07) 60 (±6.33) 30 (±5.72) 10 (±0.02)

* Values in parentheses represent the ± standard error of the mean.

**Table 2:** Mean (n #3) characteristics of the farmyard manure (FYM) used in this study.

Parameters	Units	FYM
Dry matter	(%)	95.96 (±9.82) *
Organic matter	(%)	16.92 (±1.33)
pH_H2O_ 1:5	-	8.2 (±0.10)
EC_H2O_ 1:5	(dS m^−1^)	4.2 (±0.09)
Total C	(%)	19.80 (±0.12)
N total	(%)	1.23 (±0.10)
N min	(%)	0.34 (±0.17)
C:N ratio	-	16.09

* Values in parentheses represent the ± standard error of the mean.

**Table 3:** Soil chemical parameters after 90 days of incubation. The values are the means of three replications (*n* = 3). The small letters indicate significant differences among the treatments.

Treatments	pH	EC dS m^−1^	OM (%)	TOC (%)
Clay Loam				
0	7.4 ± 0.18 ab	0.32 ± 0.02 f	1.7 ± 0.4 abcde	0.97 abcdef
ZnONPs	7.2 ± 0.06 abc	0.70 ± 0.01 cd	1.5 ± 0.3 abcdef	0.89 abcdef
FeONPs	7.2 ± 0.24 abc	0.69 ± 0.00 cd	2.1 ± 0.1 abcdef	1.20 abcdef
FYM	6.9 ± 0.06 abcd	0.88 ± 0.01 ab	3.1 ± 0.5 ab	1.78 ab
FYM+ZnONPs	7 ± 0.03 abcd	0.54 ± 0.01 e	2.7 ± 0.4 abc	1.59 abc
FYM+FeONPs	7 ± 0.12 abcd	0.55 ± 0.02 e	3.3 ± 0.2 a	1.93 a
Sandy Clay Loam				
0	6.6 ± 0.09 cd	0.37 ± 0.01 f	2.3 ± 0.9 abcdef	1.31 abcde
ZnONPs	6.8 ± 0.09 abcd	0.78 ± 0.02 bc	2.0 ± 0.2 abcdef	1.16 abcdef
FeONPs	6.4 ± 0.20 d	0.69 ± 0.00 cd	2.3 ± 0.3 abcde	1.31 abcde
FYM	6.5 ± 0.23 cd	0.93 ± 0.01 a	2.4 ± 0.1 abcd	1.39 abcd
FYM+ZnONPs	6.7 ± 0.09 cd	0.58 ± 0.01 de	3.0 ± 0.2 ab	1.74 ab
FYM+FeONPs	6.7 ± 0.07 bcd	0.61 ± 0.01 de	3.2 ± 0.6 ab	1.86 ab
Sandy Loam				
0	6.6 ± 0.09 abcd	0.2 ± 0.01 f	0.6 ± 0.1 def	0.35 def
ZnONPs	6.8 ± 0.09 a	0.79 ± 0.00 cd	0.3 ± 0.1 f	0.19 f
FeONPs	6.4 ± 0.20 cd	0.88 ± 0.09 ab	0.5 ± 0.2 ef	0.27 ef
FYM	6.5 ± 0.23 abcd	0.97 ± 0.01 a	2 ± 0.1 abcdef	1.16 abcdef
FYM+ZnONPs	6.7 ± 0.09 cd	0.58 ± 0.01 de	1.1 ± 0.3 cdef	0.62 cdef
FYM+FeONPs	6.7 ± 0.07 a	0.66 ± 0.01 cde	1.4 ± 0.2 bcdef	0.81 bcdef

## Data Availability

Data are contained within the article.
